# Editorial: Horizontal Gene Transfer Mediated Bacterial Antibiotic Resistance

**DOI:** 10.3389/fmicb.2019.01933

**Published:** 2019-08-27

**Authors:** Dongchang Sun, Katy Jeannot, Yonghong Xiao, Charles W. Knapp

**Affiliations:** ^1^College of Biotechnology and Bioengineering, Zhejiang University of Technology, Hangzhou, China; ^2^Laboratoire de Bactériologie, Centre National de Référence (CNR) de la Résistance aux Antibiotiques, Centre Hospitalier Universitaire (CHRU) de Besançon, Besançon, France; ^3^State Key Laboratory for Diagnosis & Treatment of Infectious Diseases, The First Affiliated Hospital, School of Medicine, Zhejiang University, Hangzhou, China; ^4^Department of Civil and Environmental Engineering, Centre for Water, Environment, Sustainability, and Public Health, University of Strathclyde, Glasgow, United Kingdom

**Keywords:** horizontal gene transfer, antibiotic resistance gene, multidrug resistance, plasmid, transformation, conjugation, transposition

Bacterial antibiotic resistance, especially multidrug resistance (MDR), has become a global challenge, threatening human and animal health, food, and environment safety. The number of human deaths accounted for by MDR was estimated to increase to 10 million by 2050, exceeding the number of deaths arising from cancer (WHO, 2014). Although a bacterium is able to establish antibiotic resistance through spontaneous mutation (Salverda et al., 2017), development of MDR in the bacterium would take a long time if it only relies on self-adaptive mutation. Horizontal gene transfer (HGT) allows bacteria to exchange their genetic materials (including antibiotic resistance genes, ARGs) among diverse species (Le Roux and Blokesch, 2018), greatly fostering collaboration among bacterial population in MDR development. Recent studies reveal emergence of “superbugs” that carry a number of HGT-transferred ARGs on plasmids and tolerate almost all antibiotics (Mathers et al., 2015; Wang and Sun, 2015; Malhotra-Kumar et al., 2016). These MDR plasmids are able to be further transferred to different bacterial species, creating new “superbugs” that grow in different environments. Global emergence of “superbugs” carrying MDR plasmids (e.g., NDM-1 and MCR-1) in various environmental niches (e.g., patients, animals, and soil) indicates rapid propagation of MDR among bacterial populations. Although HGT and MDR were found to be tightly linked in “superbugs,” as revealed by surveillance studies, our knowledge about how and to what extent HGT propels development of MDR under different environmental conditions remain inadequate. The 22 publications collected in the topic “Horizontal Gene Transfer Mediated Bacterial Antibiotic Resistance” show new discoveries and recent advances concerning this issue in a wide range of fields, providing a basis for collaboratively controlling MDR in the future.

ARGs have been identified in not only human (Fang et al.; LaBreck et al.; Zeng et al.) and animals, including macaques (Mannion et al.), mealworms (Osimani et al.), ducks (Sun et al.), pigs (Chi et al.), and companion animals (Wang et al.), but also plants (Chi et al.). These ARGs include *bla* variants that encode β-lactamases for degrading the newest generation of β-lactam antibiotics (Huang et al.; Chi et al.; Zeng et al.), and *mcr*-1 that confers resistance to colistin, the last line of defense (Wang et al.). Plasmids play an important role as vehicles in transferring multiple ARGs from one MDR bacterial host to another simultaneously. Reports in this collection revealed that ARGs were often identified in plasmids from MDR bacteria living in different niches (Barrón et al.; Fang et al.; Huang et al.; LaBreck et al.; Chi et al; Mannion et al.; Sun et al.; Wang et al.; Zeng et al.). To understand how plasmids have evolved, Liang et al. and Zhang et al. reported the diversification and evolution history of a certain type of plasmid (IncHI5). One study reports an example of direct fusion of two plasmids through illegitimate recombination in the host, which produced a new plasmid with ARGs derived from the two ancestor plasmids (Chi et al.). Plasmids are generally considered to be transferred through either conjugation that requires a type IV secretion system for pushing DNA from a donor cell to a recipient cell, or natural transformation that requires a DNA uptake system for pulling DNA into a recipient cell (Sun). In this topic, two reviews revealed new ways of plasmid transfer that do not use canonical DNA pulling or pushing machineries (Hasegawa et al.; Sun), highlighting diversity of HGT mechanisms in nature. Of note, both the traditional and non-traditional natural transformation mediated plasmid transfer are physiologically controlled, although different DNA uptake machineries and competence regulation circuits are employed (Sun). Moreover, in both kinds of HGT, DNA uptake is triggered by environmental stresses, which induce expression of transcriptional regulators for activating expression of DNA uptake genes (Sun). ARGs were also found in other mobile genetic elements on bacterial chromosomes (Fang et al.; Chi et al.), especially in the integrative and conjugative elements (ICEs, also known as conjugative transposons; Fang et al.).

Transposons (Tn) and Insertion Sequences (IS) are mobile genetic elements in plasmids and chromosomes. They were frequently identified in clinical settings (Sun et al.; van der Zee et al.). In this collection, transposition-mediated transfer of carbapenem and carbapenicillin resistance genes and colistin resistance gene *mcr*-1 was documented (He et al.; van der Zee et al.). A novel mobile transposon Tn6242, which contains ARGs and is flanked by IS26, was characterized in multidrug-resistant uropathogenic *Escherichia coli* ST405 (Chowdhury et al.). Besides moving ARGs, transposons can exacerbate antibiotic resistance on a different layer. By inserting into the promoter region, a transposon activates transcription of genes associated with conjugation and significantly improved conjugation frequency (Poidevin et al.). Another study revealed that insertion of the ISCR1 (Insertion Sequence Common Region) element improved expression of downstream ARGs, providing an explanation for the frequent presence of ISCR1 in the clinical setting (Lallement et al.).

ARGs were found not only in bacteria parasitizing in higher organisms, but also in basic components of our planet earth, such as soil (Chi et al.) and water (Barrón et al.; Fang et al.; Chi et al.). Although transfer of the ARG vehicle (plasmids) has been intensively investigated under laboratory conditions, it remains unclear how plasmid disseminated in our natural environment. By monitoring the dynamic of plasmid transfer in natural soil, researchers investigated transmission of a broad host range plasmid RP4 marked by *gfp* in a natural setting (Fan et al.). Their work showed that RP4 is likely to be transferred into soil bacteria of 15 phyla within 75-day (Fan et al.), providing a foundation for estimating the impact of plasmid-mediated transfer of ARGs in the soil ecosystem. To know the distribution of ARGs in the aquatic ecosystem, another study quantified the abundance of IncP-1 plasmids in samples from Orne River (Barrón et al.). Their work concluded that plasmid-mediated adhesion to particles is one of the main contributors in the formation of MGE-reservoirs in sediments, which contributes to selective enrichment process of ARGs (Barrón et al.). These studies reveal that the impact of ARG transfer to the ecosystem could be more profound than previously thought. Exploring mechanisms of plasmid-mediated ARG spread in soil and water would be crucial for controlling antibiotic resistance in the ecosystem.

In summary, articles presented in this Research Topic demonstrate benefits of using multidisciplinary approaches to deepen our knowledge about HGT-mediated bacterial MDR. These articles analyzed the formation of bacterial MDR as a result of HGT under different settings, highlighting the importance of collaboration among different fields in fighting against MDR. We thank all participating authors for their contributions and reviewers for their constructive comments, which will be the foundation for future surveillance, investigations into mechanisms and controlling strategies of HGT-mediated MDR.

## Author Contributions

All authors listed have made a substantial, direct and intellectual contribution to the work, and approved it for publication.

### Conflict of Interest Statement

The authors declare that the research was conducted in the absence of any commercial or financial relationships that could be construed as a potential conflict of interest.
